# Providing Fault Detection from Sensor Data in Complex Machines That Build the Smart City

**DOI:** 10.3390/s22020586

**Published:** 2022-01-13

**Authors:** Alberto Gascón, Roberto Casas, David Buldain, Álvaro Marco

**Affiliations:** 1 Aragon Institute of Engineering Research, University of Zaragoza, 50018 Zaragoza, Spain; algaroche@unizar.es (A.G.); buldain@unizar.es (D.B.); amarco@unizar.es (Á.M.); 2 GeoSpatium Lab S.L., Carlos Marx 6, 50015 Zaragoza, Spain

**Keywords:** fault detection, sensor data, industry 4.0, data reduction, feature analysis, feature selection, indicators, artificial neural network

## Abstract

Household appliances, climate control machines, vehicles, elevators, cash counting machines, etc., are complex machines with key contributions to the smart city. Those devices have limited memory and processing power, but they are not just actuators; they embed tens of sensors and actuators managed by several microcontrollers and microprocessors communicated by control buses. On the other hand, predictive maintenance and the capability of identifying failures to avoid greater damage of machines is becoming a topic of great relevance in Industry 4.0, and the large amount of data to be processed is a concern. This article proposes a layered methodology to enable complex machines with automatic fault detection or predictive maintenance. It presents a layered structure to perform the collection, filtering and extraction of indicators, along with their processing. The aim is to reduce the amount of data to work with, and to optimize them by generating indicators that concentrate the information provided by data. To test its applicability, a prototype of a cash counting machine has been used. With this prototype, different failure cases have been simulated by introducing defective elements. After the extraction of the indicators, using the Kullback–Liebler divergence, it has been possible to visualize the differences between the data associated with normal and failure operation. Subsequently, using a neural network, good results have been obtained, being able to correctly classify the failure in 90% of the cases. The result of this application demonstrates the proper functioning of the proposed approach in complex machines.

## 1. Introduction

Predictive maintenance is a recent technique, the result of the evolution of maintenance techniques over the years. Initially, the most commonly used maintenance systems were corrective. These systems carry out the relevant actions once the failure has occurred. With this approach, it may happen that the repair has to be postponed instead of being repaired on the spot due to a lack of readiness. Preventive maintenance, where maintenance activities are scheduled at periodic intervals to prevent component degradation, was introduced in the 1950s. However, as in the previous case, costs remain very high.

**Figure 1:**
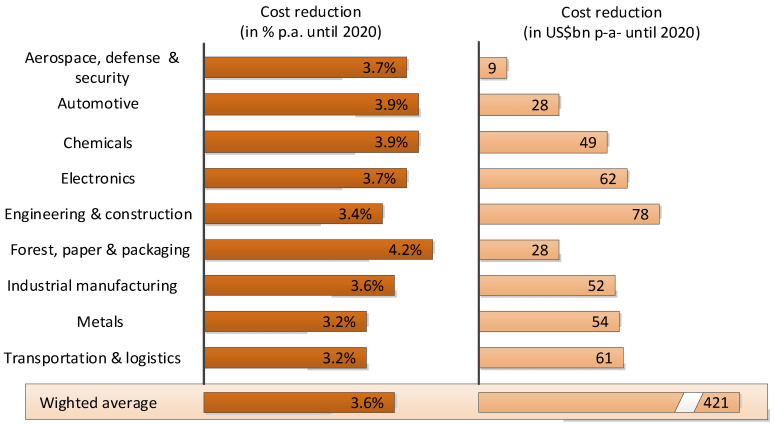
Annual cost reduction due to the incorporation of predictive maintenance techniques [1].

Given the growing demand for more reliable, safe and efficient industrial systems, the need to optimize these maintenance processes becomes evident. In the 1980s, some factories began to apply predictive maintenance techniques. They used sensors that continuously monitored the machines and sent alerts when predefined limits were exceeded. This significantly reduced scheduled maintenance activities and their associated costs. Currently, the use of large databases combined with machine learning techniques makes it possible to predict what is going to happen, when it is going to happen, and to alert the person in charge (Industrial Internet of Things, IIoT) [1,2]. In this way, a “just-in-time” maintenance that allows maximizing economic and productive performance is achieved. For this reason, as shown in Figure 1, predictive maintenance applications are having a great boom in the market, expecting, according to a PWC survey, a 3.6% reduction of the annual costs during 2020.

Predictive maintenance requires a great deal of dedication prior to installation. The problems and their causes must be identified in order to subsequently define and develop the monitoring system. This preliminary process could be structured in the following phases:

1. Detection of machines that suffer critical breakdowns for the production process.
2. Location of the machine element that produces the faults.
3. Identification of the causes that provoke the breakdowns (physical reasons why it breaks).
4. Definition of the variables to be monitored.
5. Selection of the sensors.
6. Data acquisition.
7. Data curation and extraction of indicators (features).
8. Data processing so that the system learns to detect failures.

**Figure 2:**
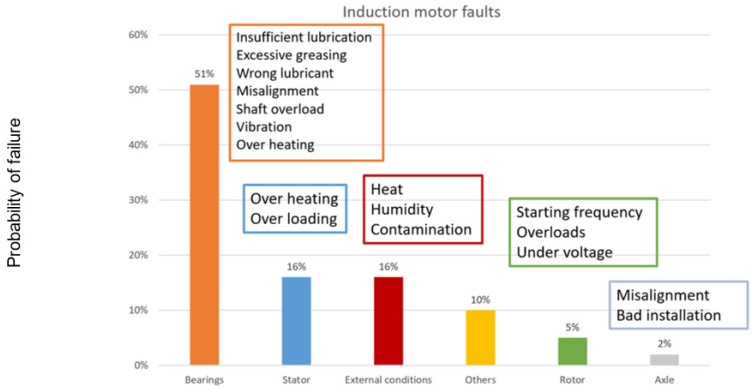
Representation of the critical elements of induction motors and their main causes of failure [3].

In industrial environments, the most relevant machines that can be found most frequently are electric motors [3,4]. Within them, the elements that concentrate the highest number of failures are the rotating elements and the transmission mechanisms due to their fatigue wear. Figure 2 shows the result of an ABB study on the critical elements and the most common causes of failure in induction electric motors.

Once the critical machines, the elements that fail most frequently and the possible causes have been identified, the subsequent phases are carried out.

In contrast to the simple sensors and actuators that make up a large part of the common Internet of Things (IoT) scenarios in the smart city [5], there is an increasing number of applications that are made up of what we may call complex machines.

**Figure 3:**
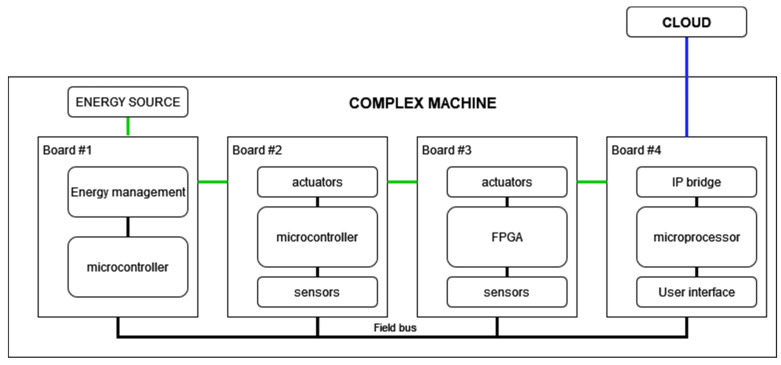
Block diagram of an example of a complex machine, green lines indicate energy flow, and blue lines indicate the data transmission to the cloud.

As illustrated in Figure 3, we define complex machine as a device that:

- Has a 24/7 operation operated by users without detailed knowledge of the operation of all the constituent parts of the machine.
- Integrates tens of sensors and actuators managed by several microcontrollers and microprocessors communicated by control buses.
- Requires energy from the mains to work, sometimes has a battery, but as a short-time backup.
- Has IP (Internet Protocols) connectivity.

Some examples of complex machines in the smart city are: household appliances, climate control machines, vehicles, elevators, cash counting machines, etc.

Technically both production lines and complex machines are made up by a network of controllers that integrate sensors and actuators. There are many works proposing predictive maintenance strategies in production lines [6] or industrial equipment [7,8]. These approaches gather all the data together in edge/fog devices [9] or in the cloud [10] and centrally analyzes them. In complex machines, this is not possible due to memory and computation restrictions of controllers and also to industrial bus bandwidth limitations.

Currently, there are different predictive maintenance strategies depending on whether they focus on physical aspects (physical model-based), on aspects of knowledge of the machine itself (knowledge-based), or if they are based on the use of large quantities data, pattern recognition, statistics, etc. (data-driven). This article proposes a fault detection methodology applicable to complex machines, trying to apply hybrid methodologies that combine the advantages of each strategy, adjusting them to the needs of complex machines. For this reason, special attention needs to be paid to preprocessing, seeking to minimize the number of data to be sent, so that malfunctions can be detected with the least amount of data possible. In this way, this strategy can be applied to machines with limited memory capacities, data transmission, etc.

This paper is organized as follows. Section 2 presents the data processing methodology and its three different levels indicating the process applied in each case: sensor level—variable targeting, board level—embedded data curation and feature extraction, and machine level—feature integration and pattern finding. Then, Section 3 illustrates the testbench used to verify the system proposed and analyzes the results obtained on each layer. Finally, Section 4 provides conclusions.

## 2. Materials and Methods

### 2.1. Data Processing Methodology

**Figure 4:**
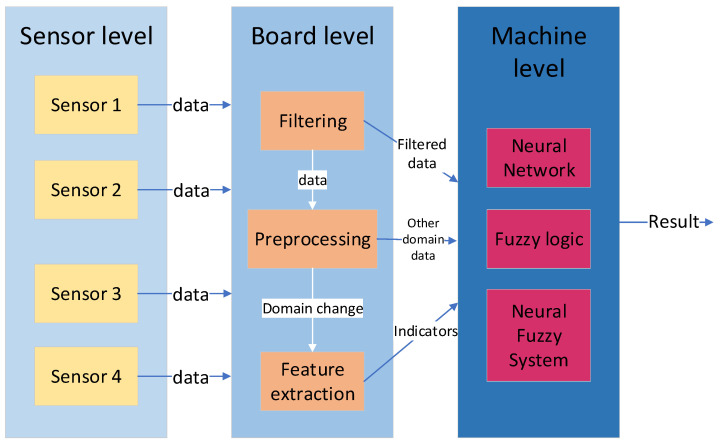
Scheme of the proposed methodology for data analysis.

A methodology for data processing consisting of three parts or levels will be proposed. The first level, called the sensor level, focuses on taking measurements using various types of sensors. The second level or board level, starts with the data obtained in the sensor level to carry out a processing that allows reducing the amount of data to be transmitted and extracting as much information as possible from them. The last level or machine level seeks to perform an analysis of the data of the board level in order to extract some results. In Figure 4, you can see the scheme of the proposed methodology.

#### 2.1.1. Sensor Level—Variable Targeting

The variables to be monitored in this type of application can be grouped into the following groups: mechanical, electrical, audio, temperature and pressure [3].

The analysis of **mechanical variables** and specifically the analysis of vibrations are the most common. Depending on the frequency range of the vibrations to be measured, position sensors (0–10 kHz), speed sensors (10 Hz–1 kHz) or accelerometers (8 Hz–15 kHz) can be used [2]. However, the use of accelerometers is the most common, as has been seen in the vast majority of the articles consulted [11,12,13,14,15,16,17,18,19]. This type of analysis presents good results, since the most common faults always generate additional vibrations to those of the engine in normal operation. Thus, through its analysis, inappropriate behavior and even the type of failure can be identified [19].

Another common approach is the analysis of **electrical variables** [4,11,13,14,15,20,21,22]. In them, the values of the stator’s motor currents and voltages are mainly monitored. The use of these variables is based on the fact that the consumptions of a damaged machine present variations compared to those of a “healthy” machine. In addition, the use of these measures has advantages, such as the possibility of measuring without having to access the interior of the motor, reducing the risk of damaging fragile parts and facilitating the installation of the sensors. On the other hand, it requires a great knowledge of the normal behavior of the machine and the different harmonics it presents due to construction characteristics or load variations. In addition, this knowledge of healthy functioning must be updated over time. Thus, applying techniques such as motor current signature analysis (MCSA), it is possible to detect anything from electrical failures, such as short circuits in the stator, to mechanical failures, such as eccentricities or rotor bar breaks [4].

In [2,23], **audio measurements** are used to detect bearing failures through the use of microphones. These types of measures are not so well-established, although they are gaining presence, as shown in [2]. The main cause is the contamination to which the audio signals are exposed in an industrial environment, requiring the use of techniques for their elimination. However, the possibility of obtaining them using microphones pointing towards the machine from the outside at between 2 and 10 cm is a clear advantage compared to vibration measurements [2].

These are the most commonly used types of measurements. However, others appear as complementary measures, such as **temperature measurements** [13,14,15,17,21] or **pressure measurements** [21], which are being used in very specific cases. A temperature increase makes possible to detect electrical and mechanical failures, since in the event of excessive friction or high electrical currents the elements tend to overheat. In addition, these types of measurements do not require complex processing, and faults can be detected by simply observing their values. Pressure measurements, for example, can be of great importance in the analysis of the motor of an air compressor (Air Booster Compressor).

#### 2.1.2. Board Level—Embedded Data Curation and Feature Extraction

Each board has a smart controller that might have wide variety of computational and memory resources; from 8-bit microcontroller to an FPGA (Field Programmable Gate Array). To extract the most relevant characteristics and reduce the volume of data used, it is necessary to perform raw data filtering or pre-processing. In the case of predictive maintenance systems, preprocessing methods can be separated into three major groups according to whether they are, in the time domain [13,14,20,21,22], in the frequency domain [4,11,15,16,17,19,23] or in the time-frequency domain [12,18].

In the **temporal domain**, an attempt is made to reduce the number of data by filtering outliers and erroneous data [14,21]. Normalization [13,21,22] becomes relevant due to the use of various variables (mechanical, electrical, temperatures, pressures, etc.) that can take values on different scales. Once the data have been adjusted, they can be used as they are [21,22], or other indicators can be extracted from the parameters. In this second case, statistical indicators (such as maximum, minimum, mean, median, standard deviation, variance, gradients, kurtosis, skewness or crest factor [14,24]) or of another type (such as the principal components [13]) can be used.

**Figure 5:**
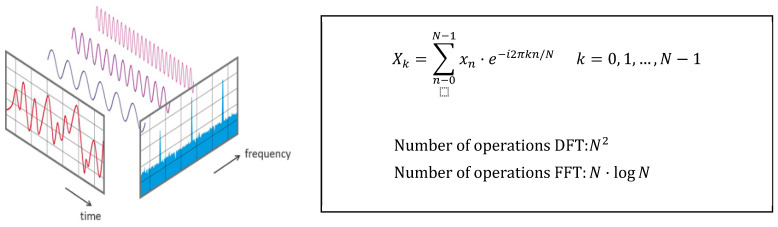
Graphical representation of the Fourier transform, equation and number of operations [25].

On the other hand, in the **frequency domain**, the vast majority of cases use the fast Fourier transform (FFT) as an analysis method [4,15,16,17,19,23]. It provides useful information through the detection of the signal’s frequency peaks and the detection of harmonics. This analysis is mainly applied when making vibration or sound wave measurements. The virtue of the FFT is that it allows decomposing a signal into individual periodic signals and establishing the relative intensity of each component, as can be seen in Figure 5. In this way, it is very easy to identify the faults, corresponding to peaks at unusual frequencies. In addition, it is a technique included in many electronic devices.

**Figure 6:**
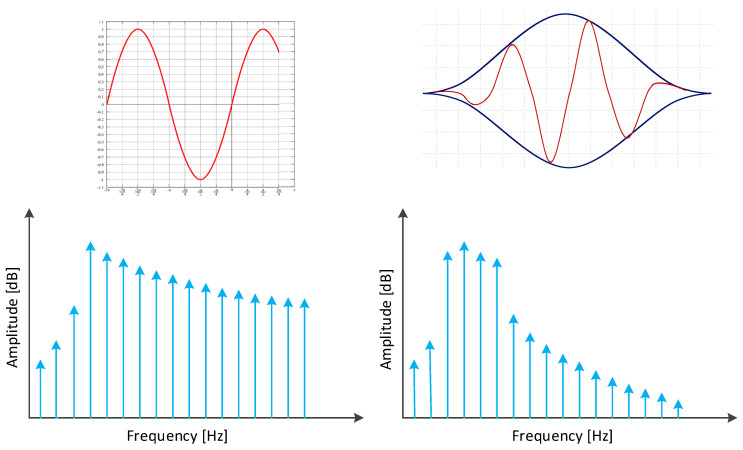
Representation of the Hanning window effect.

The FFT requires that the sampled signal contains a complete representation of the signal to be processed in the time domain or a periodic repetition. In cases where a complete cycle of the signal to be modulated is not captured, techniques such as the Hanning window are applied on the signal to mitigate possible reconstruction errors [16] (Figure 6).

Finally, techniques in the **time-frequency domain** [12,18] provide a more realistic description of the state of the machine. The main advantage they provide is that they are capable of managing both stationary and non-stationary signals (limitation presented by the FFT). The most popular for vibration analysis in rotating machines is the wavelet transform (WT) [12].

**Figure 7:**
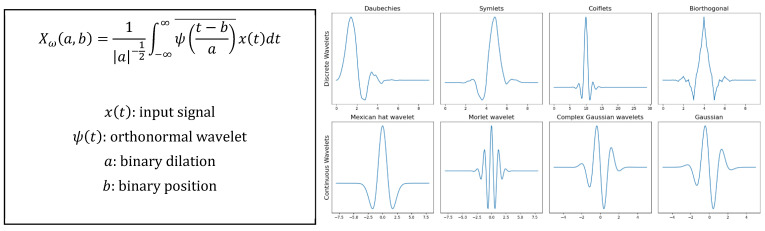
Wavelet transform equations and representation of different wavelets.

**Figure 8:**
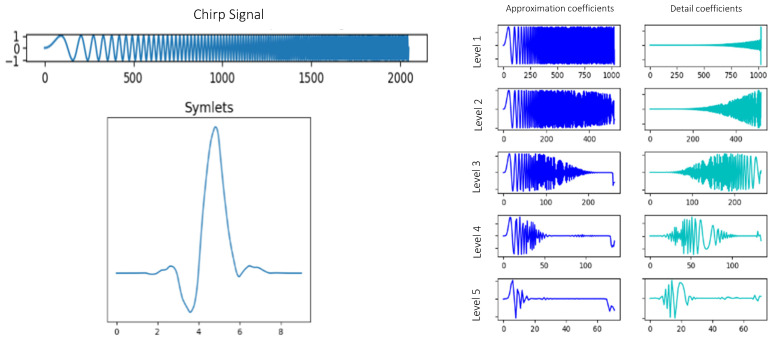
Coefficients of approximation and detail obtained by means of the WT of the signal displayed with a Symlets wavelet.

This technique starts from an orthonormal wavelet located in time that multiplies the signal. It can be applied at different times and with different scales to analyze the high and low frequency components of the signal at different points. The signal is decomposed into the approximation and detail coefficients, allowing the identification of the different frequency contributions over time. An example can be seen in Figure 7 and Figure 8.

There are also other techniques such as the Short-Time Fourier Transform, STFT [18], which consists of dividing the signal into small time windows on which the Fourier transform is applied. In this way, it is possible to know the part of the signal in which each frequency appears, but it has a lower resolution than the WT.

In the case of audio signals, an additional preprocessing would be necessary to carry out the separation of the audio signal from the ambient noise. Some of the techniques used are BSS (Blind Source Separation) or TDSEP (Temporal Decorrelation source SEParation), which allow isolating a mixture of sounds from a specific process in real time [26].

#### 2.1.3. Machine Level—Feature Integration and Pattern Finding

Once all the desired indicators have been extracted, in order to make sense of these data, it is necessary to analyze them together in what will be called the machine level. This processing would consist of the procedure for identifying possible failures. Techniques for the detection and identification of failure mechanisms are based on pattern recognition. These can be applied following complex strategies, such as the use of neural networks and machine learning [11,13,14,18,20,21,22,23], or through simpler methods, such as the use of fuzzy logic [15,16] or visual analysis by specialist personnel [17].

**Artificial neural networks** (ANN) are very convenient for this type of task, as they are able to work with a large amount of data and manage non-linearity situations with a short response time [20]. However, actual failure data are scarce, and forced failure data acquisition can be expensive. Even so, supervised learning methods are commonly used [13,18,20,21,22,23], although a predictive maintenance implementation could be initiated with unsupervised or semi-supervised learning (with labeled and unlabeled data) [14].

Finally, **simpler techniques** such as fuzzy logic are also applied. In [15,16], a classification of the data is carried out based on the ranges in which they are found, using fuzzy classifiers (good, normal, bad...). This allows a greater interpretability, something that can be tricky with neural networks. However, this apparent simplicity presents a key point that can become a bottleneck, the definition of the ranges, which requires a great knowledge of the situation to be treated. Furthermore, since it has no learning capability, it is often used in combination with neural networks, generating the so-called neural fuzzy systems (NFS) [27].

Once the results of the machine level processing have been obtained, the data can be sent to the cloud in order to perform a normality model that considers a large amount of data from different machines. Thus, while in the machines, the neural networks have patterns at the local level, in the cloud, the patterns are at a global level, which allows a greater abstraction.

**Figure 9:**
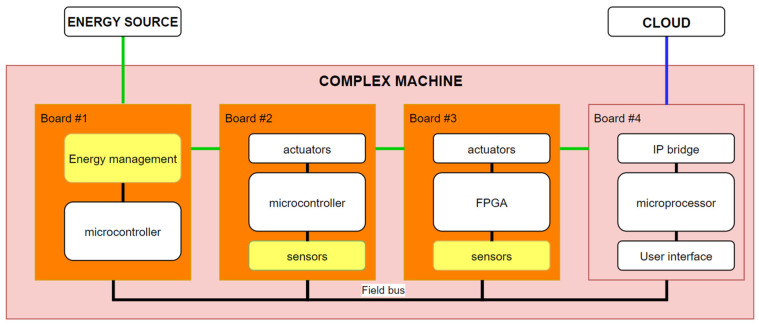
Diagram of an example of a complex machine with layer differentiation (Yellow, sensor level; orange, board level; red, machine level).

A complete example of a complex machine with the different layers can be seen represented in the diagram in Figure 9: in yellow, the lowest layer would be that of the sensor level; in orange, encompassing the previous one would be the board level; and finally, in red, encompassing the previous two, is the machine level.

In this example, it can be seen how the first three boards (energy management, microcontrollers and FPGAs with actuators) communicate with a fourth, which is differentiated by the ability to communicate with the outside. Said communication can be both, with the user (Human Machine Interface) and with another computational element, proposed in the example through an IP bridge. This board also has processing capacity, along with large RAM and FLASH memories, so that it can be in charge of analyzing the results obtained. In this way, this fourth board is in charge of receiving possible orders from the user, performing an analysis of the data received from other boards and sending the data to a downstream processing unit (in the cloud in the proposed example).

## 3. Results and Discussion

### 3.1. Testbench Definition

**Figure 10:**
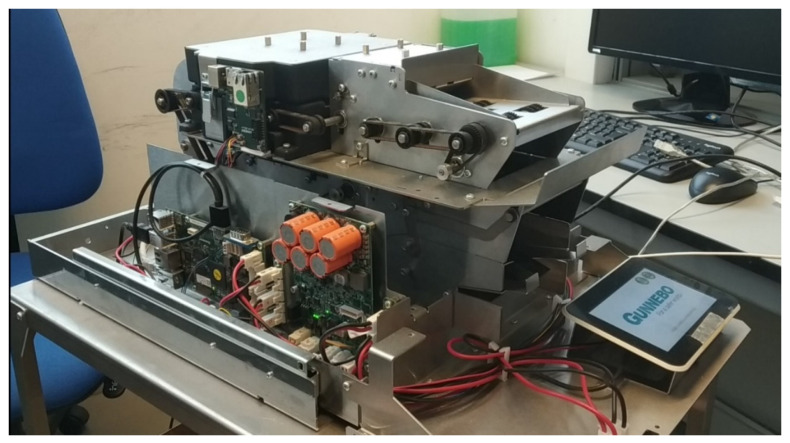
Image of the prototype used to test the methodology.

The machine on which this methodology will be applied is a machine designed to count banknotes that will be used mainly in bank branches to be able to count cash and make deposits safely (Figure 10).

**Figure 11:**
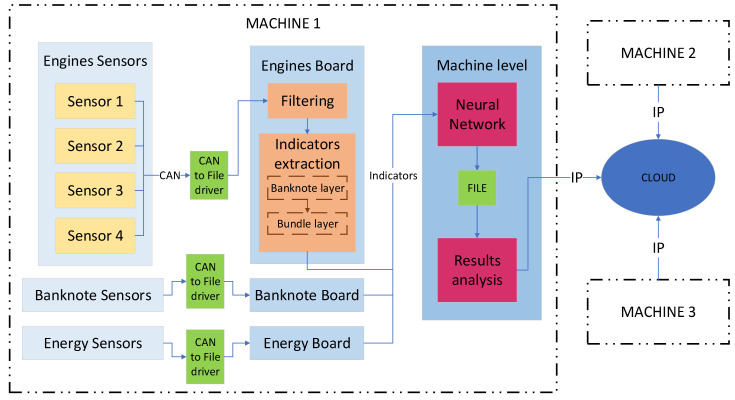
Scheme of application of the methodology to the specific case.

**Figure 12:**
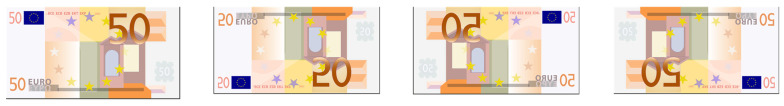
Disposition of a €50 banknote in the different orientations analyzed.

The complex machine is made up of a main board and three secondary boards: energy board, engines board and energy board (Figure 11), and the variables that are going to be monitored are shown in Table 1:

The tests will consist of passing bundles of 50 banknotes of the same denomination (5 €, 10 €, 20 € and 50 €) through the machine. In addition, each bundle will pass through the machine four times, placing all the banknotes in every possible orientation: front, back, reverse front, and reverse back (Figure 12). This results in 16 samples for each tested case, making a total of 800 banknotes analyzed per case.

Regarding the failures analyzed, 13 defects (Table 2) were forced into the machine through variations in eccentricities in axles (4) and wheels (3), use of defective components such as springs (2), dented bearings (2), and deteriorated pulleys and worn belts (2):

This means that the whole dataset consists of 11,200 banknotes records. The proposed strategy will focus on detecting failures that could be called permanent, this means that they will appear throughout all the data collection and not sporadically, something that could also happen under real operating conditions.

### 3.2. Data Analysis

#### 3.2.1. Layer 1: Sensor Data

Sensor data layer capture is accomplished by three boards, which gather the variables listed in Table 1. Once the data of the different failure cases have been obtained, they are analyzed and compared with those of the normal operation case. It is important to know the shapes and values of the data distributions in order to better understand the indicators to be extracted in subsequent layers, since this allows us to assess the best strategies to analyze them and perform a more efficient maintenance.

**Figure 13:**
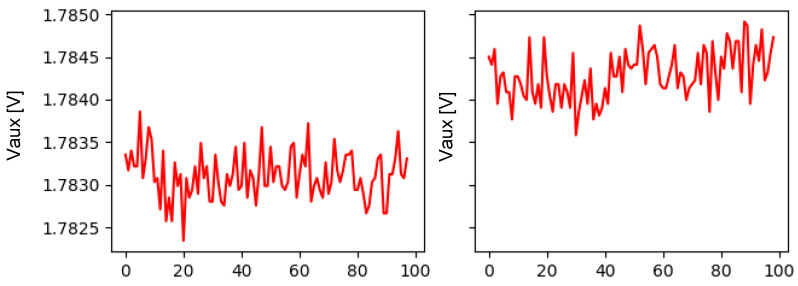
Comparison of the Vaux tension measurements of a 5 € banknote on reverse back orientation in the case of normal operation (**left**) and a failure case (**right**).

We will begin by analyzing the data obtained by the energy management board. Some of the measurements taken are voltage measurements at different points or temperature measurements, among others. When comparing the voltage data of various banknotes according to their orientation and obtained in different situations, differences can be appreciated. In Figure 13, it can be seen how the failure case shows higher values for *Vaux* than the case of normal operation. Although these are differences of a very small order of magnitude, given that the values present a very small variation, they must be taken into account.

**Figure 14:**
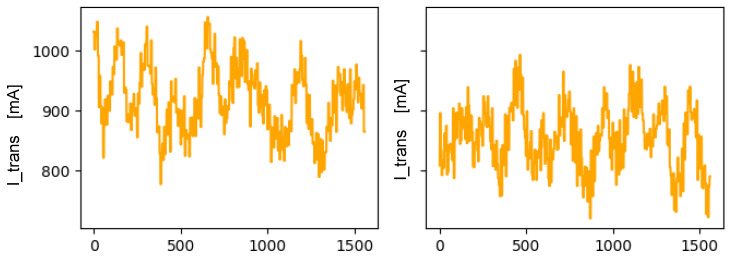
Comparison of the measurements of the transport motor current of a 50 € banknote on reverse front orientation in the case of normal operation (**left**) and that of a failure case (**right**).

The engines board has sensors that take measurements related to the mechanical operation of the machine, such as consumption of the machine’s motors or an FFT for the vibrations analysis. Figure 14 shows the current consumption during the passage of different banknotes. We can see that when introducing the modification in the machine, the current figures have been altered. Although there is still a significant overlap in the ranges, the values of the failure case present values below what would be considered normal.

**Figure 15:**
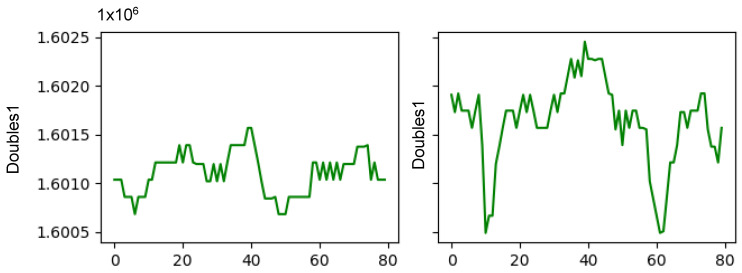
Comparison of the measurements of the double sensor 1 of a 20 € banknote on back orientation in the case of normal operation (**left**) and one case of failure (**right**).

The last of the boards included in the complex machine analyzed is responsible for monitoring the condition of the banknotes through various measurements. One of the sensors used provides values that are proportional to the thickness of the banknote passing through the machine, called the doubles sensor. Figure 15 shows the measurements obtained in the normal case versus one of the failure cases analyzed, presenting clear differences that would allow the identification of such operation as erroneous.

#### 3.2.2. Layer 2: Board Data

The amount of data provided by the machine is very high, since for each banknote (a banknote takes 610 ms to pass through the machine) 33,000 bytes would be received from the engines board, 9150 from the banknotes board and 7320 from the energy management board. Therefore, the need to reduce this number through filtering and extraction of indicators becomes evident.

In order to perform an initial filtering to reduce the number of data to be processed, it is decided to use only the data relating to the passage of a banknote through the machine. In this way, all data taken between banknotes are discarded. In addition, since the measurements of some sensors are only of interest when the banknote passes through them, it is necessary to generate a specific window for each of them. For this purpose, position sensors are used, which allow us to know the position of the banknote in the machine, being able to select the data only for those moments. Just through this filtering, we reduce to 8808 bytes per banknote from the engines board, 640 from the banknotes board and 394 from the power management board, a reduction of an order of magnitude.

**Figure 16:**
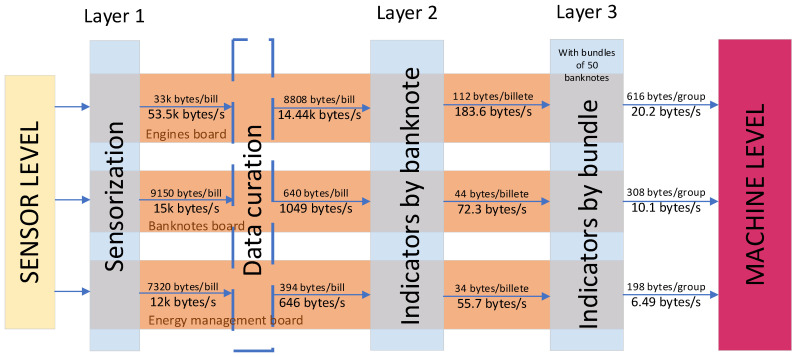
Diagram of the designed monitoring infrastructure.

When proposing the indicators to be extracted, a layered data analysis was chosen, as shown in Figure 16. The first layer would be the sensorization layer, the output of which is the raw data. After the filtering process that would follow the sensorization layer, the next layer would be that of the indicators per banknote, in which various indicators corresponding to each note are obtained. Finally, the last one would be that of the indicators per bundle, which aggregates the indicators per banknote into groups of a given number.

Observing the output data rates of the last of the layers, it can be seen that a reduction of three orders of magnitude in the bytes per second that are obtained from each board has been achieved. With this extraction of indicators, by comparing the values of the training phase with the values obtained in subsequent measurements, it will be possible to detect deviations that will allow the identification of possible failures.

The indicators per banknote to be used are, in general, the means, medians, maximums, minimums, standard deviations, asymmetries and kurtosis of the different measurements available in the frames, adding the effective value in the case of currents.

**Figure 17:**
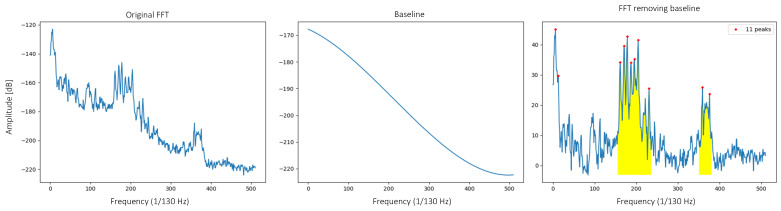
Graphs of the different phases of the identification process, from the original FFT, to the FFT without the base noise, with the detected peaks and the colored areas of interest.

Regarding the FFTs with a Hanning window (Figure 6) obtained for the vibration data, a more complex analysis is conducted. To obtain the indicators extracted in the layer of indicators per banknote, the areas under the curve of different parts of the FFT will be obtained. In order to discover the more interesting parts, we will begin by identifying the existing peaks. This identification consists of two phases: a first one in which the base noise is eliminated, leaving a flatter FFT in which the peaks stand out more; and a second one in which the peaks higher than half the maximum value are marked. Having identified the most interesting parts as those that concentrate the majority of the peaks, the areas under these parts of the FFTs will be used as the indicators of the vibration data (the limits of the areas are defined based on observation). In a preliminary analysis of the FFTs, it has been seen that in most of them, there are two areas of interest in which most of the peaks are concentrated (see Figure 17). Therefore, it is decided to work with these two areas for subsequent analysis.

Once the indicators per banknote have been extracted, they are passed to the layer of the indicators per bundle. Since the objective is the data reduction for a fault detection application, it is not sought to have an instantaneous view of the machine operation. A broader vision that allows observing the variations in a larger temporal space is more interesting. Therefore, the integration of the banknote level indicators will be done in groups of the same number of banknotes, from which indicators will be extracted per bundle. The indicators extracted from the indicators of the previous layer will be the same seven previous statistical values as above, the means, medians, maximums, minimums, the standard deviations, the skewness and the kurtosis.

#### 3.2.3. Layer 3: Machine Data

Finally, after obtaining the indicators from the bundle layer, the indicators reach the machine level. In this layer, conclusions will be drawn from the indicators extracted in the previous processes. For this purpose, this analysis seeks to identify the most relevant indicators for each failure case, as well as the type of variation that should be expected based on their probability distributions. Next, we will comment on the results obtained by comparing the distributions of the indicators of the respective failure case with those of the normal case.

The objective is to indicate whether the indicators of the failure case have higher or lower values than those of the normal case, as well as the degree of discordance between the distributions of these indicators. The indicators analyzed will be the indicators per-bundle-mean. If no specific indicator is mentioned (AVG, Med, MAX, MIN, DES, SK or KUR), the mean values are assumed to be the ones mentioned.

To assess the direction of the variation, the median of the distributions is used. On the other hand, to assess the degree of discordance, the Kullback–Leibler divergence is used. This is a unitless measure that compares the probability densities of two distributions. It provides values close to zero with two similar distributions and it grows as the difference between both distributions increases. It is not symmetric, so two calculus are made, considering first the normal case and then the failure one (P‖Q) and then viceversa (Q‖P). From these two values, the larger one is the one considered. The choice of the Kullback–Leibler divergence as a measure of comparison of the data distributions obtained in each failure case is based on the fact that the final model for failure classification will be implemented by neural networks trained with the cross-entropy cost function, which is directly related to the divergence measure. Thus, the nomenclature used is the one shown in Table 3 (limits used are based on experimental observation) and the results obtained can be seen in Table 4.

It is convenient to take the data in the summary table with caution, since there are distributions that, although they do not present divergences greater than the minimum, they do show variations with respect to the normal case.

Next, the indicators of interest for the three failure cases in group five, associated with defects in the doubles sensor (case 5), will be shown. This case of failure has been chosen because it presents deviations in a great variety of indicators.

**Figure 18:**
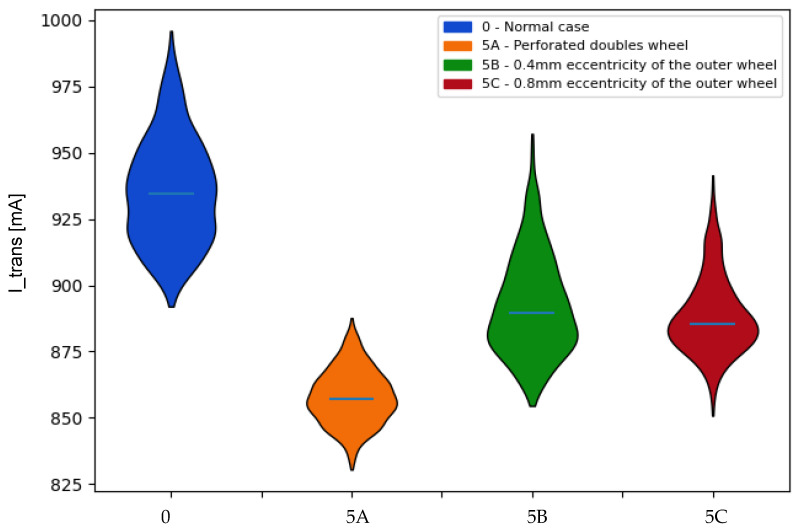
Comparison of the probability distributions obtained for the mean values of the transport motor current in the case of faults associated with the double sensors. Indicator: mean.

In the data of the **transport motor current** (Figure 18), it can be seen how in all cases of failure, the values obtained are reduced, the most notable being that of the first failure. In the following cases, in Table 5 it can be seen how the medians decrease in the distributions and the divergences increase as the eccentricity increases.

**Figure 19:**
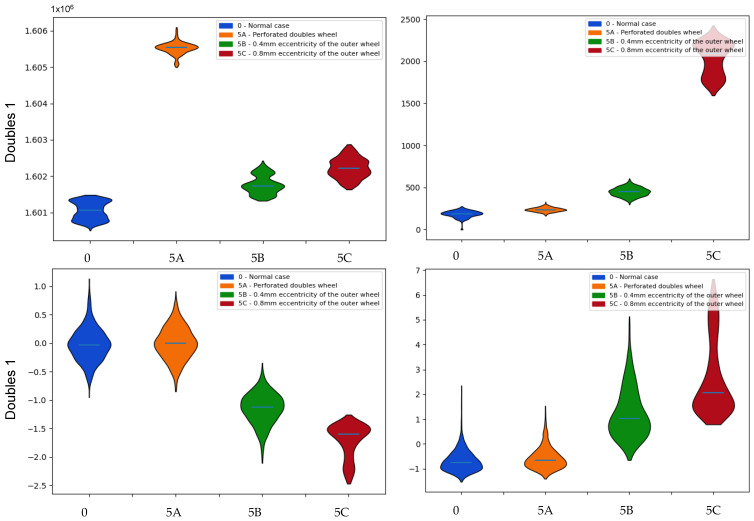
Comparison of the probability distributions obtained for the mean values of the doubles sensor 1 measurements in the case of failures associated with the doubles sensors. Indicators: (**top left**) mean, (**top right**) standard deviation, (**bottom left**) skewness and (**bottom right**) kurtosis.

The data from the **doubles sensor 1** (Figure 19) shows deviations in all three cases analyzed, something that might be expected, as the defects are introduced in the sensor itself. Regarding the average values, it is seen that the one that suffers the most divergence is the first failure of the chopped roller, while those associated with eccentricities show smaller variations, but that vary in a linear way with increasing eccentricity. Analyzing the shape statistics, it can be seen how the first failure shows values quite similar to the normal case of standard deviations and coefficients of skewness and kurtosis. However, the failures associated with eccentricities show much larger differences, highlighting the standard deviation in the case of greater eccentricity, which is more than five times higher than that of the normal case. All this can be supported by the divergence values obtained in Table 6.

**Figure 20:**
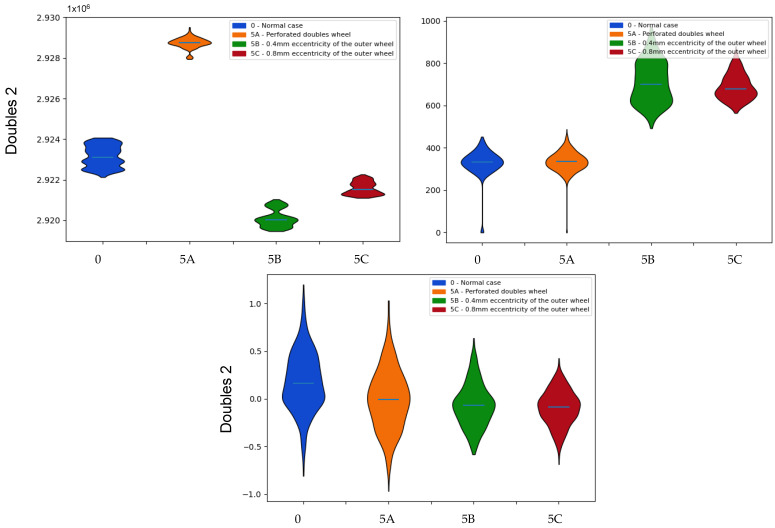
Comparison of the probability distributions obtained for the mean values of the doubles sensor 2 measurements in the case of failures associated with the doubles sensors. Indicators: (**top left**) mean, (**top right**) standard deviation, (**bottom**) skewness.

Regarding **the doubles sensor 2** (Figure 20), distributions of the mean values are very similar to those of the previous sensor. However, in the shape statistics, there are differences with respect to the previous sensor in the cases of failures associated with eccentricities. The standard deviations are no longer so far apart, although they still show considerable divergences (Table 7). The asymmetries no longer present values so far away from the normal ones, and only in the case of higher eccentricity is a significant divergence observed.

**Figure 21:**
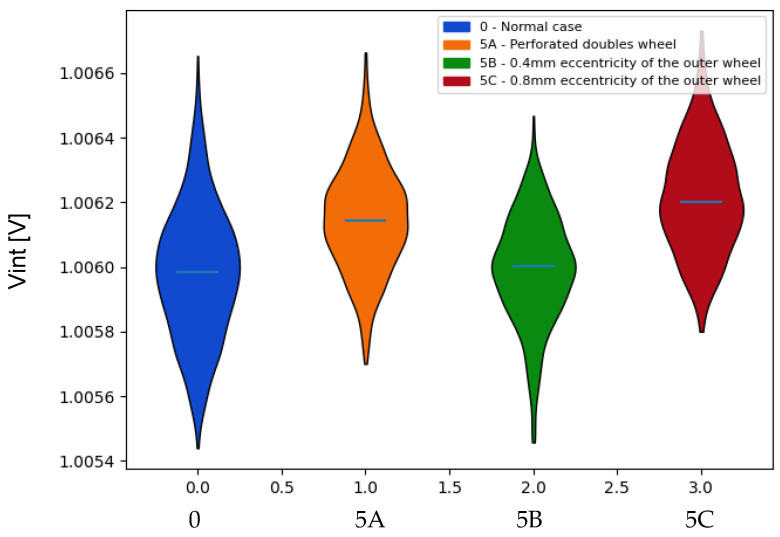
Comparison of the probability distributions obtained for the mean values of the voltage Vint in the case of failures associated with the double sensors. Indicator: mean.

Analyzing the values obtained for the Vint voltage (Figure 21), it can be seen that the second fault shows hardly any variations with respect to those of the normal case, while the other two show values higher than the usual ones. These effects are reflected in the divergences in Table 8, obtaining the highest value in the fault with the highest eccentricity.

Observing the results of this first analysis (Table 4), it is clear that some measurements such as the feed motor currents (I_feed), some infrared pass-times or the intervals between encoder pulses do not provide much information. In the case of the supply currents, it may be due to the fact that none of the altered elements affected it directly, which means that there are hardly any changes from one case to another. As for the T_IR31 and T_IR33 times, since they are associated with short distances compared to the T_IR11 time, and with sections far away from the middle zone where the defects are located, this means that they are not so easily altered. Finally, the intervals between encoder pulses present a multimodal distribution that makes the extraction of information more difficult.

By detecting these differences with the naked eye on a measure related to the cross-entropy cost function used to train the network, it is expected that the neural model in charge of processing these indicators will also be able to identify the failures. To this end, a feedforward multilayer neural network with one hidden layer was explored and, after testing several architectures, a 39:128:14 architecture was chosen. The 39 input neurons correspond to the most relevant indicators, while the 14 output neurons are associated with the 13 failure cases analyzed and the case of normal operation.

Due to the large variety of ranges present in these input variables, a normalization is performed to equalize the contributions of each variable to the multilayer perceptron. This normalization is performed before the data enters the network. Between the two processing layers that compose it, another batch normalization layer has been added with the same objective.

The processing layers that compose the model are two dense layers of 128 and 14 neurons, respectively. The first layer uses the RELU function as the activation function. In the case of the second one, there is one neuron for each class and the activation function chosen is softmax. Thus, the output of each neuron will be between 0 and 1, summing all of them 1 (generates a probability distribution), assuming that the output with the maximum value is the correct failure/normal case.

**Figure 22:**
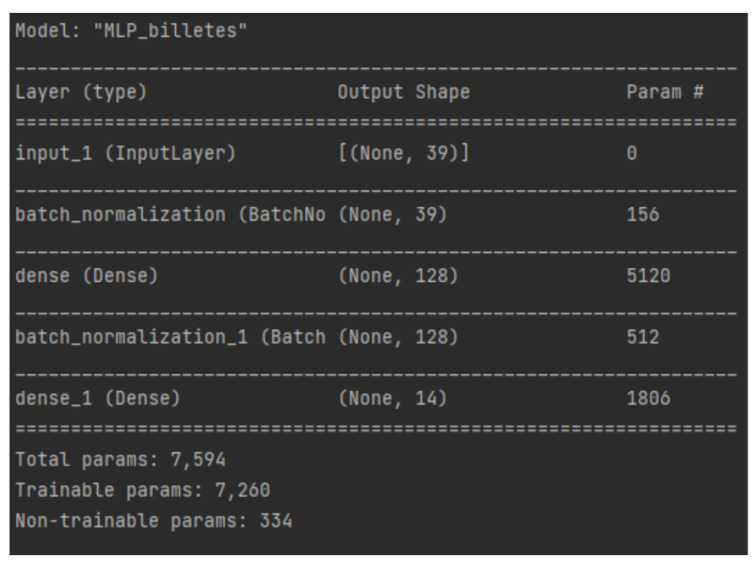
Summary of the implemented MLP.

The supervision labels follow the one-hot encoding (13 labels to zero and the corresponding class to one) and the optimization of the training hyperparameters was performed with the Adam optimization algorithm [28]. Figure 22 shows the summary of the implemented MLP architecture. Cross entropy was used as a cost function to perform the training over 40 epochs.

A cross-validation methodology was implemented to perform the training phase of several networks to generate the full model. To reduce the possibility of the data sets in the cross-validations becoming unbalanced due to sparse data, cross-validation with a low k-fold of 5 was used. In addition, to maintain data representativeness, these subsets were randomly generated using a stratified split to ensure that each test subset and training subset has statistical values of the label distribution equivalent to those of the whole dataset. In this way, five MLPs are generated, with the final model prediction being the mean or vote of the five different independent results.

**Figure 23:**
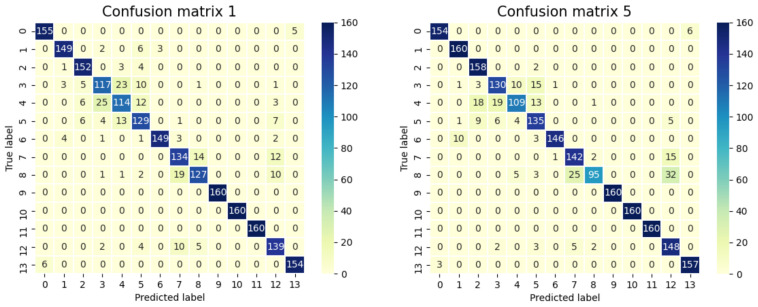
Confusion matrices of two neural networks designed to recognize the 14 failure cases.

At the end of the training, five MLPs were obtained with accuracies around 90%. However, as the classification result will be the result of the vote of the five networks, the reliability of the final model is even higher. The possibility of three networks being wrong, generating a bad prediction, would be close to 1%, which considerably increases the confidence of the classification. Figure 23 shows the confusion matrices of two of the five networks, which confirm the good performance of the methodology when applied to a specific case of fault detection on complex machinery.

Despite the good results obtained, it must be taken into account that this model has been made with data that were not isolated for validation. This has been the case because, as there is not a large amount of data available, we have worked with the available data, extracted manually by the manufacturers themselves. That is why the neural model, although showing good results and could be developed at some point, must currently be taken with caution. However, the fact that manual rule-based analysis of the results shows perceptible differences proves that the effort put into preprocessing the data to reduce the number of bytes to be sent has been successful. In this way, the objective of our proposal is reached, being able to identify the failures with a smaller amount of information and also being able to implement this methodology in complex machines with limited capacities.

## 4. Conclusions

In this work, a study of the state of the art of predictive maintenance techniques, focused on industrial environments, has been carried out. The variables to be monitored, the most relevant indicators and the most common treatment techniques have been analyzed to generate a basis on which to build the fault detection system to be carried out.

A monitoring methodology applicable to complex machines has been presented, in which work is carried out at different levels with the aim of extracting the most significant features from each piece of data. This methodology has been tested on a prototype cash counting machine, which meets the description of a complex machine.

By analyzing the results obtained in each of the levels in which we have worked, we have identified the most relevant data. In this way, it has been possible to significantly reduce the amount of data to be used, being able to continue identifying the corresponding failure in each case. All this allows a considerable reduction in the computational demand of the system.

As future work, the use of a cloud computing environment is proposed. Communication via IP with the cloud of numerous machines would allow the generation of a global neural model that reaches a higher degree of abstraction than can be achieved locally on the machine.

In short, a fault dectection system capable of identifying the failure that is occurring from the data extracted from a complex machine has been developed. This makes it posible to warn the operator who can correct the defect or it also can be used as a manufacturing quality control system.

## Figures and Tables

**Table 1:** Summary of the variables monitored.

Variable	Board	Abreviation	Unit	Bits
Transport engine current	Engines	I_trans	mA	12
Feeding engine current	Engines	I_feed		12
Transport engine encoder ticks	Engines	N_pul_trans	Number of counter ticks between two encoder pulses	12
Feeding engine encoder ticks	Engines	N_pul_feed		12
Infrarred sensor 1a	Engines	IR1a	0 no obstacle, 1 obstacle	1
Infrarred sensor 1b	Engines	IR1b		1
Infrarred sensor 2	Engines	IR2		1
Infrarred sensor 3a	Engines	IR3a		1
Infrarred sensor 3b	Engines	IR3b		1
FFT of microphone measures	Engines	FFT	-	1024
Doubles sensor 1	Banknotes	Doubles1	Measure proprtional to banknote’s thickness	32
Doubles sensor 2	Banknotes	Doubles2		32
Temperature	Energy	Temp	Celsius degrees	16
Internal voltage	Energy	Vint	V	16
Auxiliary voltage	Energy	Vaux		16

**Table 2:** Summary of the tested cases.

Identifier		Name of Failure
0	Normal operation case	
1	Effect of eccentricity in axle 2	
	A	Concentrity deviation of 0.2 mm
	B	Concentrity deviation of 0.5 mm
2	Effect of eccentricity in axle 4	
	A	Concentrity deviation of 0.2 mm.
	B	Concentrity deviation of 0.5 mm.
3	Effect of dented bearings:	
	A	Dented bearing in axle 2.
	B	Dented bearing in axle 3.
4	Effect of defective springs:	
	A	Spring without screw at BNF.
	B	Spring without screw at the entrance of the safe.
5	Effect of defective doubles sensors:	
	A	Perforated doubles wheel.
	B	Eccentricity of 0.04 mm of the outer wheel.
	C	Eccentricity of 0.08 mm of the outer wheel.
6	Deteriorated pulleys and worn belts:	
	A	Deteriorated 32 z pulley.
	B	Worn S2M 180 belt and deteriorated exit pulley.

**Table 3:** Nomenclature used to classify the differences between distributions.

Divergence	KL > 4	KL > 5	KL > 10	KL > 15
Higher	LS	S	SS	SSS
Lower	LI	I	II	III

**Table 4:** Summary of the information of the indicators of interest in each case.

Failures		1		2		3		4		5			6	
Indicators		A	B	A	B	A	B	A	B	A	B	C	A	B
Current	I_trans	SSS	=	II	II	I	=	III	III	III	II	III	III	III
	I_feed	=	=	=	=	=	=	=	=	=	=	=	=	=
Time between IR	T_IR11	=	=	S	S	=	=	=	=	=	=	=	=	=
	T_IR31	=	=	=	=	=	=	=	=	=	=	=	=	=
	T_IR33	=	=	=	=	=	=	=	=	=	=	=	=	=
N_pul_trans		=	=	=	=	=	=	=	=	=	=	=	=	=
N_pul_feed		=	=	=	=	=	=	=	=	=	=	=	=	=
Doubles sensors	Doubles 1	III	III	S	=	S	SSS	SSS	S	AVG SSS DES S	AVG SSS DES SSS SK III KUR SSS	AVG SSS DES SSS SK III KUR SSS	SS	AVG SSS DES S
	Doubles 2	AVG I DES II	AVG III DES II	S	LS	SS	SSS	SSS	S	AVG SSS	AVG III DES SSS	AVG III DES SSS SK I	SS	AVG SSS DES SSS SK SS
Voltages	Vint	=	=	=	=	=	=	=	=	=	=	LS	LS	=
	Vaux	=	=	=	=	=	S	=	=	=	=	=	=	=
FFTs	Energy 1	=	=	=	=	=	=	=	I	=	=	=	LI	=
	Energy 2	=	=	=	=	=	=	=	I	II	=	=	I	I

**Table 5:** Kullback–Leibler divergence values associated with the mean values of the transport motor current for the identification of the faults associated with the double sensors.

I_Trans	Kullback–Leibler					
	0-5A	5A-0	0-5B	5B-0	0-5C	5C-0
Mean	25.322	26.229	6.836	14.215	12.087	17.932

**Table 6:** Kullback–Leibler divergence values associated with the means, standard deviations, skewness and kurtosis associated with the mean values of the double 1 sensor measurements for the identification of the failures associated with the double sensors.

Doubles 1	Kullback–Leibler					
	0-5A	5A-0	0-5B	5B-0	0-5C	5C-0
Mean	26.985	27.402	19.838	21.173	25.869	25.379
Std.Dev.	6.509	3.008	26.270	25.759	28.101	26.075
Skewness	0.355	0.202	22.922	22.689	25.899	26.216
Kurtosis	0.297	0.469	15.573	10.018	26.375	16.953

**Table 7:** Kullback–Leibler divergence values associated with the means, standard deviations and asymmetries associated with the mean values of the double 1 sensor measurements for the identification of the failures associated with the double sensors.

Doubles 2	Kullback–Leibler					
	0-5A	5A-0	0-5B	5B-0	0-5C	5C-0
Mean	26.516	27.226	25.925	26.189	25.012	24.793
Std. Dev	0.101	0.231	26.762	25.599	26.639	26.132
Skewness	0.588	0.663	2.222	0.761	5.800	0.983

**Table 8:** Kullback–Leibler divergence values associated with the means associated with the average values of the Vint voltage for the identification of the failures associated with the double sensors.

Vint	Kullback–Leibler					
	0-5A	5A-0	0-5B	5B-0	0-5C	5C-0
Mean	2.196	0.669	0.516	0.42	4.760	1.414
